# Mechanistic/mammalian target of rapamycin and side effects of antipsychotics: insights into mechanisms and implications for therapy

**DOI:** 10.1038/s41398-021-01778-w

**Published:** 2022-01-10

**Authors:** Chuanjun Zhuo, Yong Xu, Weihong Hou, Jiayue Chen, Qianchen Li, Zhidong Liu, Guangqian Dou, Yun Sun, Ranli Li, Xiaoyan Ma, Hongjun Tian, Chunhua Zhou

**Affiliations:** 1 Key Laboratory of Multiple Organ Damages of Major Psychoses (MODMP Lab), Tianjin Fourth Center Hospital, The Fourth Central Hospital Affiliated to Nankai University, The Fourth Central Hospital Affiliated to Tianjin Medical University, Tianjin, 300140 China; 2 The First Affiliated Hospital/Zhengzhou University, Biological Psychiatry International Joint Laboratory of Henan/Zhengzhou University, Henan Psychiatric Transformation Research Key Laboratory/Zhengzhou University, Zhengzhou, 450052 China; 3 Department of Psychiatry, School of Basic Medical Science, Tianjin Medical University, Tianjin, 300075 China; 4 Psychiatric-Neuroimaging-Genetics and Comorbidity Laboratory (PNGC_Lab), Tianjin Anding Hospital, Mental Health Teaching Hospital of Tianjin Medical University Tianjin, Tianjin, 300222 China; 5 Department of Psychiatry, Tianjin Mental Health Center of Tianjin Medical University, Tianjin Anding Hospital, Tianjin, 300222 China; 6 Mental Disorder Treatment Center (MDT) of The First Affilliated Hospital of Shanxi Medical University, Taiyuan, Shanxi Province China; 7 Department of Biochemistry and Molecular Biology, Zhengzhou University, Zhengzhou, Henan 450001 China; 8 Department of Pharmacology, The First Hospital Affiliated to Hebei Medical University, Shijiazhuang, Hebei 050000 China

**Keywords:** Psychiatric disorders, Schizophrenia

## Abstract

Antipsychotic pharmacotherapy has been widely recommended as the standard of care for the treatment of acute schizophrenia and psychotic symptoms of other psychiatric disorders. However, there are growing concerns regarding antipsychotic-induced side effects, including weight gain, metabolic syndrome (MetS), and extrapyramidal motor disorders, which not only decrease patient compliance, but also predispose to diabetes and cardiovascular diseases. To date, most studies and reviews on the mechanisms of antipsychotic-induced metabolic side effects have focused on central nervous system mediation of appetite and food intake. However, disturbance in glucose and lipid metabolism, and hepatic steatosis induced by antipsychotic drugs might precede weight gain and MetS. Recent studies have demonstrated that the mechanistic/mammalian target of rapamycin (mTOR) pathway plays a critical regulatory role in the pathophysiology of antipsychotic drug-induced disorders of hepatic glucose and lipid metabolism. Furthermore, antipsychotic drugs promote striatal mTOR pathway activation that contributes to extrapyramidal motor side effects. Although recent findings have advanced the understanding of the role of the mTOR pathway in antipsychotic-induced side effects, few reviews have been conducted on this emerging topic. In this review, we synthesize key findings by focusing on the roles of the hepatic and striatal mTOR pathways in the pathogenesis of metabolic and extrapyramidal side effects, respectively. We further discuss the potential therapeutic benefits of normalizing excessive mTOR pathway activation with mTOR specific inhibitors. A deeper understanding of pathogenesis may inform future intervention strategies using the pharmacological or genetic inhibitors of mTOR to prevent and manage antipsychotic-induced side effects.

## Introduction

Antipsychotic pharmacotherapy is recommended as the standard of care by all major international guidelines for the treatment of acute schizophrenia, and is considered an effective option in the management of psychotic symptoms caused by other psychiatric disorders [1, 2]. Despite the clinical efficacy of both first- and second-generation antipsychotic drugs, there are growing concerns regarding their side effects that include metabolic and neurological sequelae. For example, olanzapine, one of the most effective second-generation antipsychotic drugs, induces weight gain within weeks of the initiation of therapy and causes adverse metabolic effects that persist throughout the entire treatment course. Side effects decrease patient adherence and diminish the clinical utility of antipsychotic drugs. Clearly, insights into the mechanisms that underlie the side effects of antipsychotic drugs are needed.

The mechanisms of antipsychotic drug toxicity remain largely unknown. Heretofore, most studies of antipsychotic-induced weight gain and metabolic syndrome (MetS) have focused on the central nervous system (CNS) mediation of appetite and increased food intake [3, 4]. Nevertheless, antipsychotic drugs may dysregulate glucose and lipid metabolism and promote hepatic steatosis, thus predisposing to weight gain and MetS [5, 6]. Recent studies to elucidate the mechanisms underlying antipsychotic drug-induced metabolic and neurological adverse effects suggest that the mechanistic/mammalian target of rapamycin (mTOR) pathway plays a critical regulatory role in the pathophysiology of these disorders. Furthermore, antipsychotic drugs activate the mTOR pathway in the striatum, which is comprised of approximately 95% medium spiny neurons (MSNs) that have a key role in the cerebral control of motor function and behavior, and thereby contribute to the development of extrapyramidal side effects. Although important advances have been made in the understanding of the role of the mTOR pathway in antipsychotic-induced side effects, few reviews have been conducted on this emerging topic. We thus attempted to synthesize key findings that indicate a pivotal role of the hepatic mTOR pathway in the pathogenesis of metabolic complications, and that implicate dysregulated striatal mTOR signaling as a major etiology of extrapyramidal motor adverse effects. For example, Ramirez-Jarquin and colleagues recently demonstrated that the striatal mTOR pathway plays a direct role in the extrapyramidal motor side effects of haloperidol, a typical first-generation antipsychotic drug widely prescribed to treat patients suffering from schizophrenia or other psychiatric diseases [7]. Both pharmacological inhibition of the mTOR complex 1 (mTORC1) by rapamycin and *mTOR* gene silencing abrogated haloperidol-induced extrapyramidal side effects in a murine model [7]. An increasing number of studies have linked extrapyramidal movement disorders to excessive activation of the mTOR signaling pathway in MSNs that leads to both upregulated protein biosynthesis and suppression of autophagy that causes accumulation of toxic proteins [7–9]. These findings suggest that normalization of activated striatal mTOR signaling with specific inhibitors of mTOR may prevent extrapyramidal side effects caused by dopamine 2 receptor (D2R) antagonists [7, 9].

Although progress has been made in elucidating the role of the mTOR pathway in the pathogenesis of antipsychotic-induced side effects, questions remain to be answered. For example, how does dysregulation of mTOR signaling lead to dysfunction of striatal MSNs and the consequent clinical phenotype? Why is the activation of mTOR signaling dependent on D2R? Insights into pathogenesis are expected to pave new roads for medical interventions to mitigate antipsychotic drug-induced metabolic and neurological side effects. However, there are challenges in translating novel research discoveries into future clinical applications.

In this review, we aimed to synthesize recent key findings regarding the hepatic and striatal mTOR pathways in the pathogenesis of metabolic and extrapyramidal side effects, respectively. Furthermore, we discuss implications of normalizing aberrant mTOR pathway activation through pharmacologic and genetic approaches to prevent and manage antipsychotic-induced metabolic and neurologic side effects.

## Side effects of antipsychotic drugs

Antipsychotic drugs are associated with metabolic and extrapyramidal motor complications, hyperprolactinemia, and non-neurological/non-metabolic (e.g., cardiovascular and anticholinergic) side effects (NNSEs).

### Metabolic side effects

Antipsychotic medications have long been known to exert metabolic side effects, primarily comprising disturbances in glucose and lipid metabolism, leading to obesity, insulin resistance, prediabetes, diabetes, MetS, dyslipidemia, and other metabolic disorders [10]. Consequently, these metabolic conditions predispose to other diseases [e.g., cardiovascular disorders (CVD), stroke] that may shorten the life-span of schizophrenia patients receiving long-term antipsychotic therapy [10, 11]. For example, two widely prescribed second-generation antipsychotic drugs, olanzapine and clozapine, have been frequently reported to cause MetS, and have been strongly linked to dyslipidemia [12, 13]. Clozapine and olanzapine significantly increased fasting plasma glucose and lipid levels, promoted histologically confirmed hepatic steatosis, and induced glucose metabolic disorders in rat models [14–16]. Of note, the adverse effects of clozapine and olanzapine were time-dependent [14] and dose-dependent [15]. At present, clozapine is the only evidence-based, approved antipsychotic treatment for patients with treatment-resistant schizophrenia (TRS). In clinical practice, switching to clozapine is highly recommended for the treatment of TRS patients [17, 18]. However, the risk of metabolic adverse events, especially diabetes, among TRS patients is of increasing concern [19–22]. To date, few investigations have assessed clozapine-induced MetS in TRS patients. Most recently, we performed a clinical study to evaluate clozapine-induced prediabetes/diabetes in the treatment of schizophrenia patients with the early-treatment resistance (E-TR) subtype. Notably, of 230 schizophrenia patients with the E-TR subtype, 170 and 6 patients developed clozapine-induced prediabetes and diabetes, respectively, accounting for 76.52% of the studied subjects [23]. Collectively, these previous studies, including ours, have demonstrated that the incidence of clozapine-induced prediabetes/diabetes and other metabolic side effects is considerably high.

Pérez-Iglesias and colleagues studied the course of metabolic abnormalities and weight gain following first-treated psychotic episodes. Patients were randomly assigned to receive either olanzapine, haloperidol, or risperidone. During the study period of up to 3 years, the first year was the most critical period for weight gain and other metabolic abnormalities associated with increased CVD risk [24]. De Hert and colleagues examined metabolic parameters of drug-naive schizophrenic patients, and found that most weight gain occurred within the first few months of antipsychotic therapy [10]. In an analysis of 86 clinical trials, Millen and colleagues reported that the greatest weight gain in adults receiving oral or depot formulations of olanzapine occurred during the first three months of therapy, and continued afterwards at a reduced rate, with a plateau reached at 6–12 months [25].

Other studies have assessed metabolic parameters and abnormalities in patients receiving a variety of antipsychotic agents [26, 27]. For example, Feng et al. [26]. compared differences in metabolic side effect profiles between long-term olanzapine versus clozapine treatment for schizophrenia or schizoaffective disorder. Of note, a significantly larger proportion of olanzapine recipients developed diabetes during the study period of up to eight years. To date, no comprehensive comparative studies have addressed whether and how antipsychotic-induced metabolic side effects differ between medium- and long-term therapy. Most recently, Schneider-Thoma et al. [28] reported a protocol for conducting a meta-analysis of randomized controlled trials of antipsychotic drug-associated metabolic side effects (primarily weight gain and disturbances of glucose and lipid metabolism) in schizophrenia patients during medium- to long-term treatment. Their systematic review is presumably ongoing; no results have been reported to date. In summary, published studies indicate that antipsychotic medications cause metabolic side effects, with the greatest effect observed in the short-term, ranging from several months to 1 year after the initiation of therapy.

Huhn and colleagues performed a systematic review and network meta-analysis of 402 randomized controlled clinical trials on the efficacy and side effects of antipsychotic drugs in schizophrenia patients [29]. Forty-six percent (12/26) of antipsychotic drugs caused significantly more weight gain compared with placebo [29]. The mean weight gain was most different between the second-generation antipsychotic zotepine [associated with the most weight gain [3.21 kg, 95% conference of interval ((CI), 2.1–4.31)] and the first-generation antipsychotic haloperidol [associated with the least weight gain (0.54 kg, 95%CI, 0.15–0.95)]. In addition to zotepine, the other two second-generation antipsychotics, olanzapine and sertindole, induced significantly more weight gain than most of the other drugs^2^9. The newer class of antipsychotic agents (e.g., zotepine, olanzapine, and sertindole) caused more pronounced metabolic side effects than first-generation medications.

### Extrapyramidal motor side effects

Extrapyramidal side effects of antipsychotic drugs include parkinson-like disturbances, dystonia, dyskinesia, tremor, rigidity, and bradykinesia, and akathisia. First-generation agents are associated with more severe and frequent adverse events [20, 29–31].

The aforementioned systematic review and network meta-analysis conducted by Huhn et al. [29] retrieved data from studies of 53,463 adult schizophrenia patients taking either oral antipsychotic drug or placebo, and aimed to rank antipsychotic drugs on the basis of their efficacy and side effects. Forty-seven percent (24,911/53,463) of patients required antiparkinsonian medications to treat extra-pyramidal movement disorders. Further analysis revealed that 66% (21/32) of antipsychotic drugs significantly increased the use of antiparkinsonian drugs in placebo-controlled trials. The need for antiparkinsonian therapy was most highly associated with haloperidol, followed by chlorpromazine, zotepine, lurasidone, risperidone, ziprasidone, paliperidone, brexpiprazole, iloperidone, amisulpride, thioridazine, aripiprazole, asenapine, quetiapine, and olanzapine. Forty-eight percent (25,783/53,463) of patients taking oral antipsychotic drugs developed akathisia. Further analysis revealed that 67% (20/30) of antipsychotic drugs significantly elevated the risk of akathisia in comparison with placebo; the risk ranking was similar to that for the use of antiparkinsonian drugs. The Huhn network meta-analysis of large data sets from randomized controlled clinical trials, together with other studies, clearly indicates that extrapyramidal side effects occur often in patients taking antipsychotic mediations, with more frequent and severe side effects associated with first-generation agents [20, 29–31]. Indeed, a number of newly developed, second-generation antipsychotic drugs (e.g., risperidone, olanzapine, zotepine, and sertindole) exert lower extrapyramidal side effects compared with first-generation agents [29, 30].

### Non-neurological/non-metabolic side effects

Antipsychotic drugs cause NNSEs such as cardiovascular abnormalities (e.g., QT interval prolongation), anticholinergic side effects, and sexual dysfunction. According to the above-mentioned network meta-analysis of Huhn et al. [29], 50% (7/14) of antipsychotic drugs caused significant QT interval prolongation compared with placebo. The mean QT interval prolongation differed substantially between antipsychotic drugs, with the least change (3.43 ms) associated with the second-generation antipsychotic quetiapine and the most pronounced effect (23.90 ms) caused by the second-generation antipsychotic sertindole [29]. Based on a comparative analysis conducted by Peluso et al. [32] and the Huhn meta-analysis, second-generation antipsychotic drugs are more likely to cause cardiovascular disorders and anticholinergic and sexual side effects [29].

## Mechanistic/mammalian target of rapamycin

### Biology of mTOR and its associated protein complexes

**Fig. 1:**
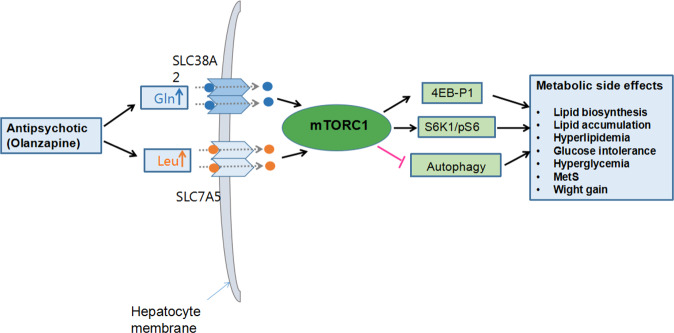
Proposed mechanisms of antipsychotic-induced metabolic side effects. Antipsychotic drugs (e.g., olanzapine) increase plasma levels of l-glutamine (Gln) and l-leucine (Leu), two validated activators of the mTOR pathway. Gln and Leu are transported into hepatocytes by amino acid transporter solute carrier (SLC) SLC38A2 and SLC7A5, respectively. High levels of Gln and Leu cause a pseudo-fasted state, and activate the hepatic mTOR pathway. Activation of mTORC1 disturbs lipid and glucose metabolism, leading to upregulated lipid synthesis, accumulation of triglycerides, storage of nutrients, hyperlipidemia, glucose intolerance, weight gain, MetS, and other metabolic side effects. 4EBP1 eukaryotic initiation factor 4E-binding protein 1, Gln l-glutamine, Leu l-Leucine, mTORC1 the mammalian target of rapamycin complex 1, MetS metabolic syndrome, pS6 ribosomal protein S6, p70s6K/S6K1 p70S6 kinase, SLC38A2 solute carrier 38A2, SLC7A5 solute carrier 7A5.

mTOR is a ubiquitously expressed serine/threonine protein kinase [molecular weight 289 kDa)] in the family of phosphoinositide 3-kinase (PI3K)-related protein kinases, and is encoded by the *MTOR* gene located in chromosome 1p36 [33, 34]. Through interacting with its protein partners, mTOR protein kinase forms two structurally and functionally distinct protein complexes: mTOR complex 1 (mTORC1) and mTOR complex 2 (mTORC2) [33–35]. A schematic illustration of the structural assembly of the two complexes is provided in Fig. 1. The protein subunits of mTORC1 include mTOR, regulatory-associated protein of mTOR (RAPTOR), proline-rich AKT1 substrate 1 (PRAS1), DEP domain-containing mTOR-interacting protein (DEPTOR), and target of rapamycin complex subunit LST8 (mLST8) [36]. As a core component of mTORC1 (mTOR-mLST8-RAPTOR), RAPTOR plays an essential role in recruiting substrates and in the subcellular localization of mTORC1, while mLST8 stabilizes the kinase domain of mTOR. In contrast to the structural assembly of mTORC1, mTORC2 comprises the following subunits: mTOR, rapamycin-insensitive companion of mTOR (RICTOR), DEPTOR, mLST8, and target of rapamycin complex 2 subunit mapkap1 (MAPKAP1), with mTOR, mLST8, and RICTOR as the core of the complex (mTOR–mLST8–RICTOR) [36, 37].

mTORC1 is found in multiple subcellular locations, including the nucleus, cytoplasm, and endoplasmic reticulum (ER). Mammalian mTORC2 was first reported in 2004 [38], and its different versions have different subcellular locations. Both mTORC1 and mTORC2 regulate cellular processes and differences as shown in Fig. 1. mTORC1 is better understood than mTORC2, primarily because of studies utilizing rapamycin, a natural compound that inhibits the yeast homolog of mTORC1 but not that of mTORC2. However, rapamycin inhibits the assembly of mTORC2 in mammals; consequently, rapamycin therapy may suppress both mTORC1 and mTORC2 signaling in humans [39, 40].

### mTOR signaling pathway in the maintenance of metabolic and energy homeostasis

More than two decades of research have yielded substantial evidence of critical roles of the mTOR signaling pathway in the maintenance of metabolic and energy homeostasis. Following activation of mTOR, cells enter into a growth mode, in which mTORC1 and mTORC2 orchestrate a wide range of diverse functions during embryonic development, aging, and diseases (e.g. cancer, diabetes, neurodegenerative disorders) by initiating biosynthetic cascades [33, 41, 42]. Here, we review the distinct roles of mTORC1 and mTORC2.

### Roles of mTORC1

mTORC1 catalyzes its substrates and subsequent effectors, promoting the biosynthesis of lipids, proteins, nucleotides, and ATP; but reducing the autophagic breakdown of cellular components. mTORC1 has four major functions:

(1) Activation of 5′ cap-dependent translation and protein synthesis. mTORC1 tightly regulates 5′ cap-dependent translation and protein synthesis by phosphorylating p70S6 kinase (S6K1) and eukaryotic initiation factor 4E-binding protein 1 (4E-BP1), an essential component of the eukaryotic translation initiation factor 4E (eIF4E) cap-binding complex [43–45]. As a result of mTOR-mediated phosphorylation of 4E-BP1, eIF4E is released from the cap-binding complex, allowing 5′ cap-dependent translation of mRNAs and protein synthesis [43–45]. mTORC1 phosphorylates S6K1 which subsequently acts on its substrate ribosomal protein S6, leading to phosphorylation of S6, an essential component of the 40S subunit. mTORC1-mediated S6K1 phosphorylation activates eIF4B, which enhances cap-dependent translation and protein synthesis [46].

(2) Promotion of lipid synthesis and accumulation. mTORC1 promotes lipid synthesis and accumulation primarily through two axes involving two transcription factors: peroxisome proliferator-activated receptor-γ (PPARγ) and sterol regulatory element-binding protein 1/2 (SREBP1/2). mTORC1 promotes the translocation of PPARγ and SREBP1/2 to the nucleus where these transcription factors up-regulate the expression of genes that encode lipid synthesis [47, 48].

(3) Enhancement of glycolysis and energy production. mTORC1 directly activates transcription factor hypoxia inducible factor 1α (HIF1α), through which it enhances glycolysis to alter the metabolic fate of glucose, and to facilitate energy production via oxidative phosphorylation [49, 50].

(4) Suppression of autophagy. mTORC1 plays an inhibitory role in the regulation of autophagy through two effectors, autophagy-related protein 13 (ATG13) and unc-51-like autophagy-activating kinase 1 (ULK1) that are required for the formation of autophagosomes [51–53]. mTOR, by phosphorylating ULK1 and ATG13, suppresses autophagy [51–53].

### Roles of mTORC2

Compared to mTORC1, mTORC2 is poorly understood; its direct effectors and substrates remain largely unknown. mTORC2 phosphorylates protein kinases in the AGC kinase family, consisting of members of cAMP-dependent protein kinases A, cGMP-dependent protein kinases G, and phospholipid-dependent protein kinases C [e.g., Akt, also known as protein kinase B (PKB); and protein kinase C (PKC)]. mTORC2 phosphorylates the serine/threonine protein kinase Akt on Ser473, and exerts a central role in the regulation of glucose, lipid, and amino acid metabolism [54–57].

### Dysregulation of the mTOR pathway in human diseases

Because the mTOR signaling plays pivotal roles in diverse cellular processes, it is not surprising that dysregulation of this fundamental pathway has been associated with the pathogenesis of a broad range of human diseases (e.g., cancers, metabolic, and neurodevelopmental disorders) [50, 58].

## Roles of mTOR signaling in the pathogenesis of antipsychotic-induced side effects

mTORC1 and mTORC2 have key roles in the signal transduction of major antipsychotics; consequently, the mTOR signaling pathway may contribute to pathogenesis of side effects. Here, we discuss the role of mTOR signaling in the pathogenesis of anti-psychotic drug-induced metabolic and neurological side effects.

### mTOR pathway in the pathogenesis of antipsychotic drug-induced metabolic side effects

A growing body of evidence suggests that hepatic mTOR signaling plays an important role in the mediation of anti-psychotic drug-induced metabolic side effects. Schmidt et al. [6]. demonstrated that olanzapine up-regulates hepatic AMP-activated protein kinase (AMPK) signaling that promoted glucose and lipid metabolic disorders in a murine model. Olanzapine-treated mice displayed significantly increased body weight, liver-to-body weight ratio, and fat pad mass; however, a commensurate increase in food intake was not observed, suggesting that these side effects may not be directly attributable to the CNS-mediated enhancement of appetite and food intake. Further metabolomic analysis revealed that olanzapine-induced anaerobic glycolysis and a pseudo-fasted state, which in turn depleted hepatic glycogen reserves in a murine model and in an in vitro HepG2 cell culture system [6]. OLZ concomitantly upregulated the mTOR pathway and downstream signaling cascades in mice. mTOR pathway activation was demonstrated by several lines of evidence, including increased mTOR phosphorylation at Ser248, S6K at Thr389, and 4EBP1; and activation of mTORC1 [6].

In two previous studies, enhanced mTOR activity disrupted hepatic lipid homeostasis by regulating the expression of sterol regulatory element-binding protein-1c (SREBP-1c) transcription factor [59, 60]. Congruent with this report, other studies found that olanzapine significantly increased SREBP-1c expression, which was suppressed by inhibiting mTORC1 activity with rapamycin [6, 16]. These studies suggest that olanzapine simultaneously activates the mTOR and AMPK pathways that may mediate metabolic complications, and provide evidence that the hepatic mTOR signaling pathway has a central role in the pathogenesis of antipsychotic drug-induced metabolic side effects.

Olanzapine can increase peripheral blood levels of l-glutamine (Gln), Gln metabolites, and l-leucine (Leu) [61, 62]. Certain amino acids (AAs) (e.g., Leu, arginine, Gln) activate hepatic S6K through mTOR/mTORC1 signaling to sense nutrients in cultured hepatocytes and other cell types [36, 63, 64]. Gln and Leu are brought through the hepatocyte cell membrane by L-type amino acid transporters in the solute carrier (SLC) superfamily (e.g., SLC38A2, SLC7A5). Under conditions of high blood concentrations of Gln and Leu, Gln in combination with Leu directly activate the mTOR pathway [65]. Jewell et al. [66]. have demonstrated that mTORC1 is differentially activated by Gln and Leu, and that RagA and RagB are required for Leu-mediated activation of mTORC1. The adenosine diphosphate ribosylation factor-1 GTPase is essential for Gln-induced activation of mTORC1 and its subsequent lysosomal localization. Building upon these findings, we proposed the potential mechanisms through which the hepatic mTOR pathway is activated by high hepatocellular concentrations of Gln and Leu, and suggest that excessive hepatic mTOR signaling may mediate antipsychotic drug-induced metabolic side effects [41, 67]. As illustrated in Fig. 1, we propose that olanzapine increases blood levels of Gln and Leu, which are carried by specific amino acid transporters [solute carrier (SLC) SLC38A2 for Gln and SLC7A5 for Leu] into hepatocytes. Hepatic mTOR signaling senses elevated Gln and Leu levels as a pseudo-fasted state and is aberrantly activated. mTORC1 activation disturbs lipid and glucose metabolism, leading to upregulated lipid biosynthesis and accumulation of triglycerides (TG). In addition, activation of the mTOR pathway suppresses autophagy and thereby increases intracellular lipid accumulation. Through multiple mTOR-mediated pathways, antipsychotic drugs induce hyperlipidemia, glucose intolerance, weight gain, MetS, and other metabolic side effects.

### mTOR signaling in the pathogenesis of antipsychotic drug-induced neurological side effects

In contrast to the numerous studies elucidating the roles of the mTOR signaling pathway in antipsychotic-induced metabolic side effects, relatively few investigations have explored its potential role in the pathogenesis of neurological sequelae. However, it has become increasingly evident that mTOR signaling plays an important part in extrapyramidal motor disorders caused by antipsychotic drugs.

Haloperidol, as a dopamine 2 receptor (D2R) antagonist, is a typical first-generation antipsychotic drug commonly prescribed to treat psychosis in patients with schizophrenia [68, 69]. Despite its high clinical efficacy, haloperidol causes severe extrapyramidal motor side effects in a majority of patients. These neurological complications have limited its clinical use substantially [69]. The mechanisms underlying haloperidol-induced extrapyramidal side effects are still undetermined. Most recently, a research team from the Scripps Research Institute investigated the brain tissue-specific roles of the *mTOR* gene in murine models using genetic and pharmacological approaches [7]. Their results indicate that striatal mTOR signaling directly modulates motor behaviors and mediates haloperidol-induced extrapyramidal side effects [7]. Ramirez-Jarquin et al. [7]. examined the role of the striatal mTOR pathway in regulating motor behaviors using conditional silencing of the *mTOR* gene in mTOR^flox/flox^ mice. Their results provided clear evidence to support the direct regulatory role of striatal mTOR signaling in motor behavior under normal conditions. Furthermore, mTOR gene depletion in the striatum abrogated haloperidol-induced extrapyramidal side effects [7].

Dopamine regulates motor function through the promotion of two major classes of receptors in the striatum, dopamine 1 receptor and dopamine 2 receptor (D1R and D2R, respectively) [70]. Of note, *mTOR* gene depletion in the striatum did not alter the extrapyramidal motor effects of SCH23390, a D1R antagonist. In contrast, depletion of the striatal *mTOR* gene abrogated D2R-mediated motor effects without altering D1R-mediated movement disorders in mice [7]. Given that haloperidol acts as a D2R antagonist, these results suggest a specific role of mTOR signaling in the pathogenesis of haloperidol-induced extrapyramidal motor side effects. Pretreatment with haloperidol at a dose of 0.5 mg/kg (i.p.) inhibited post-synaptic D2R signaling and robustly elicited extrapyramidal motor disorders in control mice. However, haloperidol failed to trigger extrapyramidal movement disorders in the mTOR-depleted mice. Moreover, haloperidol failed to promote striatal phosphorylation of ribosomal protein pS6 (S235/236) in mTOR-depleted mice compared with the control mice with the WT *mTOR* gene. In agreement with the findings in *mTOR* depleted mice, pretreatment with rapamycin completely abolished haloperidol-triggered extrapyramidal side effects in a time-dependent manner [7].

Mechanistic studies of pharmacological inhibition of mTOR activity using rapamycin disclosed that haloperidol-induced striatal ribosomal protein pS6 (S235/236) and pGluR1 (S845) signaling, while pharmacological inhibition with rapamycin and striatal mTOR depletion diminished S6K as well as phosphorylation levels of ribosomal pS6 without interference with pGluR1 induced by haloperidol in C57BL/6 WT mice. These findings suggest that pharmacological antagonism of mTOR by rapamycin suppresses haloperidol-promoted mTORC1 signaling in the striatum, thus reducing haloperidol-induced extrapyramidal side effects [7].

**Fig. 2:**
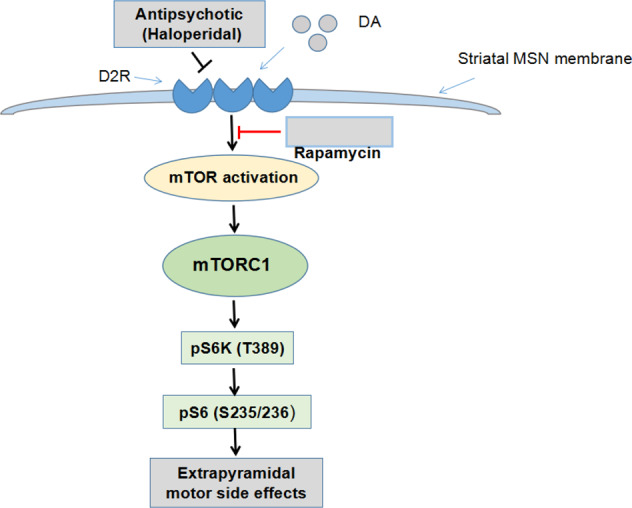
Proposed mechanism of haloperidol-induced extrapyramidal side effects through the striatal mTOR/S6K axis. Haloperidol, as a pure dopamine 2 receptor (D2R) antagonist, competitively blocks post-synaptic D2R in the striatum through an unidentified effector to promote mTOR pathway activation. Haloperidol-induced activation of mTORC1, rather than mTORC2, acts on its target downstream effectors ribosomal protein S6 kinase (S6K) and phosphorylation ribosomal protein (pS6) to promote translation and protein biosynthesis in medium spiny neurons (MSNs), alter spine density, and cause extrapyramidal motor disorders. The mTOR/S6K axis may translate D2R antagonist (e.g., haloperidol)-mediated signaling into extrapyramidal side effects. DA dopamine, D2R dopamine 2 receptor, mTOR the mammalian target of rapamycin, mTORC1 the mammalian target of rapamycin complex 1, MSNs medium spiny neurons, pS6 ribosomal protein S6, S6K ribosomal protein S6 kinase.

Antipsychotics, including haloperidol, stimulate the phosphorylation of ribosomal pS6; however, the link between phosphorylated pS6 and cataleptic behaviors is still unclear [71, 72]. Ribosomal S6 kinase (S6K), the first AGC kinase identified as an important downstream effector of mTOR, is activated in the mTOR/S6K axis to enhance the phosphorylation of ribosomal pS6 [72, 73]. Ramirez-Jarquin et al. demonstrated that silencing of the striatal *mTOR* gene and pharmacological inhibition using rapamycin abrogated haloperidol-induced phosphorylation of ribosomal pS6 in the striatum in a murine model [7]. On the basis of these findings, we hypothesize that the mTOR/S6K axis may serve as important pathway in the pathogenesis of haloperidol-induced extrapyramidal motor side effects (Fig. 2). Collectively, the findings suggest that striatal *mTOR* and mTORC1 blockade contributes to haloperidol-induced extrapyramidal dysfunction through the mTOR/S6K axis, and that targeting the *mTOR* gene and mTORC1 may benefit patients receiving D2R antagonists.

## Implications for therapy and challenges in the management of antipsychotic drug side effects

The association of excessive mTOR signaling to the metabolic and neurological side effects of antipsychotic drugs suggests potential therapeutic benefits of targeting the mTOR pathway and its downstream effectors to prevent adverse events. Aberrant activation of the mTOR pathway may be normalized by pharmacological or genetic inhibitors of mTOR. Therefore, mTOR inhibitors are proposed for not only for normalizing the hyper-activated mTOR pathway, but also for promoting autophagy, which may also mitigate antipsychotic drug toxicity.

Over the last decade, mTOR inhibitors, including the natural compound rapamycin and its synthetic analogs (e.g. temsirolimus, everolimus, ridaforolimus), have been developed for cancer therapy and immune modulation [8]. In addition, natural compounds derived from traditional Chinese medicine have been identified as mTOR inhibitors, including picrasidine M from *Picrasma quassioides*, psychotrine from *Alangium lamarckii*, and acerosin from *Vitex negundo* [74]. However, some of these mTOR inhibitors exhibit limited blood–brain barrier penetration. Therefore, the selection of mTOR inhibitor candidates for drug development for the prevention of antipsychotic-induced extrapyramidal motor side effects must consider their CNS bioavailability. Additionally, the side effect profiles of antipsychotic medications are more varied than their clinical efficacies [29], which may be attributable to divergent and complex side effect mechanisms. For example, current findings indicate a direct role of the mTOR pathway in the modulation of haloperidol-associated molecular, morphological, and functional alterations of striatal MSNs [7, 72, 75, 76]. It may merit attention that haloperidol-associated activation of the mTOR pathway is D2R-dependent without evidence of D1R involvement [7]. The mTOR pathway could play a role in extrapyramidal motor side effects caused by D2R antagonists (e.g., haloperidol) with similar mechanisms of action on D2R rather than other dopamine receptors. Given that the mTOR pathway can regulate autophagy, additional studies to determine whether haloperidol-induced mTOR pathway activation could further reduce autophagy and whether multiple downstream pathways contribute to extrapyramidal motor side effects caused by haloperidol and similar antipsychotic drugs may be valuable. With respect to the role of the hepatic mTOR pathway in the pathogenesis of olanzapine-associated disturbances of glucose and lipid metabolism, correcting the over-activation of mTOR signaling may hypothetically prevent weight gain, obesity, prediabetes, diabetes, and other metabolic complications. In addition, in vivo and clinical studies have raised concerns regarding off-target effects of mTOR inhibitors. Going forward, studies to address these challenges are necessary.

Although mechanistic studies have paved ways to develop new pharmacologic interventions and have offered rationale for conducting clinical trials of mTOR inhibitors to reduce antipsychotic-related adverse events, much work remains to be done before these research findings can be translated into clinical applications. Challenges will include dose determination and identification of the optimal timing of therapy to prevent and manage antipsychotic drug-induced metabolic and neurological side effects. In addition, cytochrome P450 (CYP) system has been shown to play key roles in the metabolism of antipsychotic drugs (CYP2D6 and CYP3A4 for risperidone, CYP1A2 and CYP2D6 for olanzapine, CYP3A4 for quetiapine and ziprasidone, and CYP1A2 for clozapine) [77, 78]. Individuals who carry the unfavorable genotypes of CYP2D6, CYP3A4, or CYP1A2 may be susceptible to antipsychotic-induced side effects. Future well-designed studies are needed to validate these findings in antipsychotic response as well as side effects.

Despite substantial progress in the understanding of the relationship between the mTOR pathway and metabolic and extrapyramidal side effects, limitations should be acknowledged. Firstly, key effectors remain to be identified. Further in-depth mechanistic studies are still needed to fill substantial knowledge gaps. Elucidation of these mechanisms may guide the development of new approaches for the prevention of antipsychotic-induced side effects. Secondly, genetic variants in the mTOR pathway have been identified with the single nucleotide polymorphisms (SNPs) rs7211818 and rs9674559 showing a relationship with the risk of type 2 diabetes mellitus (T2DM) [79]. Yin et al. [80] reported a genetic correlation (SNPs rs2494746 or rs2494738) of the PI3K/Akt/mTOR pathway with an increasing risk of obesity and T2DM in Chinese population. Although these genetic factors have been reported to be associated with the susceptibility to obesity and diabetes, it remains unexplored about their relationship with antipsychotic-induced side effects. Therefore, it would be interesting to investigate whether individuals carrying these genetic factors on the mTOR pathway could be at higher risk of developing antipsychotic-induced metabolic side effects. With respect to epigenetics factors on the mTOR pathway, demethylation of H4K20me1 by an epigenetic regulator, namely plant homeodomain finger protein 8 (*Phf8*), has been found to result in transcriptional suppression of RSK1 and epigenetic disruption of the mTOR signaling [81]. With the interesting findings, it would be worth exploring whether the epigenetic regulation of the mTOR signaling could be involved in antipsychotic-induced side effects.

In conclusion, multiple lines of evidence indicate that hepatic mTOR signaling is a critical pathway in the antipsychotic-induced glucose and lipid metabolism, which may contribute to weight gain, MetS and other complications. Antipsychotic-induced activation of the hepatic mTOR pathway could be mediated by two specific AAs, Gln, and Leu. In addition, antipsychotic drugs promote the mTOR pathway activation in MSNs through currently unidentified effectors. With the existing studies, we postulated that sustained activation of the mTOR/S6K axis and suppressed autophagy may lead to excessive protein synthesis and toxic protein accumulation in the striatum, which may contribute to extrapyramidal movement disorders [7, 8, 82, 83]. The link between the mTOR pathway and antipsychotic-induced side effects suggests that normalizing aberrant mTOR pathway activation with specific pharmacologic or genetic inhibitors of mTOR may benefit patients in need of antipsychotic medications through the prevention and management of antipsychotic-induced side effects, enhanced treatment adherence, and improved long-term clinical outcomes.
